# The influence of prepaid service and promotion purchase restriction on consumers’ willingness to share in tourism and hospitality: from the perspective of framing effect theory

**DOI:** 10.3389/fpsyg.2022.1022312

**Published:** 2022-09-26

**Authors:** Haohan Luo, Ningning Pan, Yalin Zhong, Haijun Wang

**Affiliations:** ^1^School of Finance, Southwestern University of Finance and Economics, Chengdu, China; ^2^Faculty of Geography, Yunnan Normal University, Kunming, China; ^3^School of Economics, Beijing WUZI University, Beijing, China

**Keywords:** prepaid service, promotion purchase restrictions, perceived scarcity, perceived certainty, willingness to share, framing effect

## Abstract

Prepaid service is not only a financial tool, but also a common promotion mode in tourism and hospitality. Due to the limited resources of the enterprise, the enterprise needs to reasonably allocate the promotion resources to maximize the effectiveness of the promotion. As two common promotion purchase restrictions, limited-time promotion and limited-quantity promotion how to interact with prepaid services in the form of discounts or freebies to enhance consumers’ willingness to share is the focus of this study. This study carried out three experiments based on framing effect theory, stimulus-organism-response theory, and social capital theory, which has found that the prepaid service mode moderates the relationship between promotion purchase restrictions and consumers’ willingness to share. When the prepaid service mode is a discount type, the limited-quantity promotion can generate higher sharing willingness than the limited-time promotion, and the perceived scarcity plays a mediating role. When the prepaid service mode is a freebie type, the limited-time promotion can generate higher sharing willingness than the limited-quantity promotion, and the perceived certainty of opportunity plays a mediating role.

## Introduction

Affected by COVID-19 and Internet information technology, the tourism and hospitality are placing greater emphasis on enhancing consumers’ desire to buy and share by offering “prepaid services.” In terms of cross-period allocation of funds, prepaid service is a financial service, which has the function to improve the effectiveness of both supply and demand for funds. Prepaid service is beneficial for the suppliers of products or services to obtain funds in advance to reduce financial stress, and help them understand the demand of consumers, reasonable arrangement of resource allocation, and better control of costs. Prepaid service also allows consumers to get certain service value at a low price in the present (in the form of a discount) or a higher service value for the same price in the future (in the form of a freebie). For consumers, these two options represent the “money-saving” and “value-added” features of prepayment, respectively. From the psychological and marketing perspective, prepaid service is a common promotion mode (Qiu et al., 2022), helping to increase consumers’ willingness to buy and share, which in turn increases the promotion of the product and the business performance of the suppliers. In general, enterprises providing prepaid services will attach certain promotion purchase restrictions. It is of great theoretical and practical research value to study how the interaction between prepaid service mode and promotion purchase restriction affects consumers’ willingness to share.

Different promotion modes will have different effects on consumers’ decision-making behavior (Shi et al., 2005), and under different conditions and circumstances, discount promotions with money-saving functions and freebie promotions with value-added functions will have differentiated mechanisms for consumers’ purchase willingness. Most studies have explored the influence of promotions on purchase decisions from the perspective of promotion frequency, promotion depth, promotion restrictions, product types, and individual differences among consumers (Campbell and Diamond, 1990; Kalwani and Yim, 1992; Mela et al., 1997; Sinha and Smith, 2000; Kim and Kramer, 2006; Xia and Monroe, 2009; Rodrigues et al., 2021; Wang et al., 2021; Gao et al., 2022). But few studies have paid attention to consumers’ willingness to share promotion activities. However, consumers’ willingness to share and their sharing behaviors are of even greater value to companies offering promotions, and this value is even more evident in the Internet era for the tourism and hospitality, which are affected by COVID-19. Promotions such as prepaid service offered by companies to stimulate consumers’ willingness to buy directly. But more importantly, they promote products and services through promotion activities, so that more consumers will know about the product or service, as well as the company. Because whether consumers accept promotion activities is affected by many factors, they may not be able to buy directly for themselves due to the time limitations and budget constraints, but their sharing behavior is almost unlimited in the Internet era. For enterprises, the more consumers share the promotion, the more consumers will know about the activity, which will indirectly increase the number of sales. Therefore, this study focuses on consumers’ willingness to share promotions under two different prepaid service modes, which are prevalent in the tourism and hospitality.

The resources of the enterprise are limited to offer promotions sustainably over the long term. When promotions become the norm, they can no longer be called promotions. Most promotions are short-term incentives, used to stimulate consumers to buy products or service quickly in large quantities in a relatively short period (Blattberg and Neslin, 1990). This is the reason why enterprise generally limit promotion activities. And the implementation of promotion purchase restrictions will affect consumers’ cognition and attitude to a certain extent, producing the effect of “hunger marketing.” In emphasizing limited availability, promotion purchase restrictions often include time restriction and quantity restrictions (Cialdini and James, 2009). Enterprises in the tourism and hospitality need to be in business for the long term, and the products or services provided by them are more general, which makes it difficult for them to carry out more discounted promotions by falsely increasing the unit price. This is why companies in the tourism and hospitality usually opt for real limited-time or limited-quantity promotions. As a result of the framing effect, consumers may have different perceptions of these two different ways of promotion purchase restrictions, leading to different decision-making behaviors. Many studies show that limited-quantity promotions generate higher purchase intent (Aggarwal et al., 2011), but this relationship is also affected by other variables, such as promotion modes, product types, etc. Recent studies on consumer purchase intentions are less likely to directly compare differences in the effects of different promotion modes or promotion purchase restrictions. And few studies focused on the influence of promotion purchase restrictions on consumers’ sharing behavior. For this reason, this study focuses on the influence of the interaction between the prepaid service modes and promotion purchase restrictions on the sharing willingness of consumers.

This study uses the cognitive generated by consumers under the framing effect to explore consumers’ willingness to share different types of promotion purchase restrictions under different prepaid service modes, and attempts to explain the psychological mechanisms through stimulus-organism-response theory (SOR theory for short). In theory, this study is perfection and supplement to the theoretical system of promotion; in the practice, it also provides targeted guidance and suggestions for enterprises in the tourism and hospitality to implement prepaid services and promotions.

## Literature review and hypothesis

### Framing effect of promotion type

The framing effect refers to the phenomenon where presenting the same information in different ways causes people to have preferences reversed (Tversky and Kahneman, 1981). The framing effect was first discovered and proposed by Tversky and Kahneman in their research on the “Asian Disease Problem.” This theory suggests that people’s decision-making behavior is not rational entirely, and the same decision-making problem described in different perspectives will lead to cognitive biases, which in turn affect their decision-making behavior. Since then, the framing effect theory has been widely used in the researches of psychology, finance, marketing, organizational behavior, and other fields. In tourism and hospitality, the framing effect also exists widely. Wen et al. (2021) explored the effect of option framing on travellers’ purchase decisions regarding customized travel packages. The promotion framework involved in this study is also an application of the framing effect theory (Wang et al., 2021). The framing effect theory states that when a decision problem presents a positive aspect, individuals tend to be certain, and when it presents a negative aspect, individuals tend to prefer risk. Diamond and Johnson (1990) combined with the theory of mental accounts and pointed out that, promotions are classified into gain-gaining promotions (Framed as gains) and loss-reducing promotions (Framed as reduced losses). Discount frames are those that offer consumers the same category of products at a lower price, and freebie frames are those that offer consumers a greater number of similar and other products at the same price (DelVecchio et al., 2007; Mishra and Mishra, 2011). Prepaid service in the form of freebie is less likely to be linked to the cost of the product or service and provides an additional benefit to the consumer, so it can be considered as a revenue-gaining promotion. Discounted prepaid service is considered a loss-reduction promotion because it is linked to the actual cost of the product or service (Diamond, 1992).

The existing researches on the framing effect of promotion modes mainly focus on the influence on consumers’ purchase intention, and there are inconsistent research conclusions. Diamond and Sanyal (1990) found that although freebie promotion and discount promotion are essentially equivalent promotions, consumers prefer freebie promotion. At the same time, discount promotion is more susceptible to marginal diminishing effects than freebies (Nunes and Park, 2003), and is influenced by negative background information (Chandran and Morwitz, 2006). However, contrary researches have also found that freebie promotion has a negative influence on consumer decisions. Such as freebie promotion will reduce consumers’ willingness to pay for the key products and the freebies (Kamins et al., 2009). And may have a negative influence on the brand image and brand attitude of the main product (Simonson et al., 1994). As research progresses, more and more scholars have found that different promotion modes can have different effects on consumers’ decision-making behaviors under different conditions and circumstances. Therefore, variables such as types of promotional product, frequency of promotion, depth of promotion, promotion restrictions, and individual consumer differences are gradually being included in researches to explore how their interaction with promotion modes affects consumers’ purchasing decisions (Sinha and Smith, 2000; Kim and Kramer, 2006; Xia and Monroe, 2009; Rodrigues et al., 2021; Wang et al., 2021; Gao et al., 2022). For example, Sinha and Smith (2000) found that discount promotions are more effective for low-storable products than freebie promotions, which have a better influence on fast moving consumer goods (FMCG for short) sales than discount promotions (Li et al., 2007); research by Campbell and Diamond (1990) showed that consumers are more likely to accept freebie promotions when the promotion benefits are higher; Winterich et al. (2015) found that consumers’ self-construction will affect their preferences for the promotion modes, consumers who are independent and self-constructed prefer discount promotions, while consumers who rely on self-construction prefer donation promotions. Xia and Monroe (2009) proposed that whether consumers have shopping goals will have an influence on the promotion framework, and the study found that consumers with shopping goals are more likely to accept discount promotions. However, in general, few studies have focusd on the research of consumers’ willingness to share promotion activities under different promotion modes. This study argues that consumers’ behaviors in purchasing promotional products and their behaviors in sharing promotion information are not entirely consistent. For example, consumers may find that a product in promotion is not suitable for them leading to a refusal to buy it, but the thought that the product might be suitable for a friend leads to sharing behaviors. And for enterprises, enhancing consumers’ willingness to share is one of the promotion objectives. So, this study focuses on the influence of the framing effect of promotion type on the willingness to share.

### Promotion purchase restrictions

Promotion is a temporary and short-lived activity essentially. As a promotion activity, prepaid service could play a role in promoting sales if it has certain purchase restrictions. In emphasizing limited availability, promotion purchase restrictions usually include time limits and quantity limits (Cialdini and James, 2009), it also includes membership status restrictions, monetary restrictions, etc. (Wang et al., 2021). In practice, in addition to stimulating direct purchases by consumers, promotion purchase restrictions are also an effective way for companies to promote themselves, attract consumers ‘attention, and raise their reputation. In academics, existing researches on promotion purchase restrictions have focused on the influence on consumer purchase behavior (Oruc, 2015).

From the perspective of the mechanism, the promotion purchase restriction mainly affects consumers’ purchase behaviors through two aspects. One is to stimulate consumers to make quick decisions emotionally, and promotion purchase restrictions can stimulate consumer impulse purchase behavior by affecting consumers’ perceived arousal (Corso et al., 2012; Guo et al., 2017). The second is to highlight the scarcity of promotional products to increase consumer perceived value (Cialdini and James, 2009). Previous researches have shown that limited-quantity promotions are more effective than limited-time promotions in general shopping situations because limited promotions lead to stronger perceived scarcity (Aggarwal et al., 2011). Due to the limited number of promotional products in limited-quantity promotions, there is competition with others, which makes it easier for consumers to have a psychology of competitive buying (Aggarwal et al., 2011). The state of competition will trigger stronger behavioral motivation for consumers, and the scarcity will speed up the purchase decision process and enhance purchase intention (Worchel et al., 1975). Competition with others creates a sense of tension and excitement for consumers, and the successful acquisition of a promotional product satisfies people’s desire to possess the product and gives them the satisfaction and joy of winning. Sometimes people care more about the thrill of winning the competition than the product, which can also enhance the perceived value of the consumer (Babin et al., 1994). Taken together, the researches have shown that limited-quantity promotions are generally better at driving consumer purchase behaviors than limited-time promotions. However, this effect is also affected by other factors, such as the moderating of product types, the moderating of promotion modes, the moderating of individual uniqueness needs, etc. (Gierl et al., 2008; Jang et al., 2015; Wang et al., 2021). Gierl et al. (2008) found that conspicuous consumption products are better suited to the use of limited-quantity scarcity signals rather than limited-time scarcity signals. Jang et al. (2015) found that limited-time scarcity information can increase consumers’ purchase intention for non-limited-edition products, while limited-edition scarcity behaviors can positively influence people’s purchase intention for limited-edition products. Wang et al. (2021) suggested that the promotion mode moderates the relationship between promotion purchase restrictions and consumer purchase intention. Specifically, when the promotion mode is a freebie type, limited-time promotions generate higher purchase intention compared to limited-quantity promotions. When the promotion mode is a discount type, limited-quantity promotion can generate higher purchase intention than limited-time promotion.

In the Internet information era, the mutual influence between consumers has been greatly enhanced. The information released by the companies must become a topic of discussion among consumers and be shared by them to achieve the best dissemination effect. Economic stimulus not only increases consumers’ purchasing behaviors, but also has positively affects on word-of-mouth communication. However, few researches have focused on the influence of promotion purchase restrictions on consumers’ willingness to share and its mechanism, which is particularly important for companies to improve promotion efficiency.

### The SOR theory and hypothesis

Prepaid service is offered as a discount or as a freebie usually, representing a “money-saving feature” and a “value-added feature” respectively. In the practice of the tourism or hospitality, prepaid service is often used in combination with limited-time or limited-quantity promotions, two common forms of promotion purchase restrictions. Is the willingness to share triggered by limited-time promotions and limited-quantity promotions consistent across different prepaid service scenarios? This study intends to use the SOR theory to explore the influence and mechanism of promotion purchase restrictions on consumers’ willingness to share. According to the SOR theory, the stimulus is an external influence that can affect people’s psychological state, which in turn prompts people to respond (Namkung and Jang, 2010). Stimulation affects the mind through the consciousness of the recipient, who is the organism. After being stimulated, it forms a conscious or unconscious psychological response of an organism, the mental state at this time can be either an emotional state or a cognitive state (Jacoby, 2002). After a series of psychological response processes, the recipient will adopt an internal or external behavioral response to the stimulus. Intrinsic responses are usually attitudes, while behavioral responses are usually approach or avoidance behavior (Eroglu et al., 2003). The SOR theory has been applied to the study of the influence of promotions on consumer purchasing behavior, such as Gao et al. (2022) built a livestreaming impulsive buying model based on the SOR theory, and they explored the influence of sales promotion on impulsive buying. This study considers prepaid services and promotion purchase restrictions as external incentives given by companies to consumers; the perceived certainty of opportunities and perceived scarcity is the psychological perception of consumers, which is a psychological state formed by external stimulus, which in turn generates sharing willingness (internal response to the stimulus of promotion information) and sharing behavior (external behavioral response to promotion information stimulus).

### Discounted prepaid service scenarios

The discounted prepaid service mode has the function of saving money for consumers. Because this promotion mode reduces the actual payment of the consumer, which is directly related to the product price and this will emphasize how much the consumer needs to spend, and the loss information will be highlighted. According to the theory of framing effects, the loss of framing information leads individuals to establish higher demands, that is, to pursue higher values (Wang, 2002; Mishra and Fiddick, 2012). Individuals are sensitive to loss information, so people generally tend to avoid losses. According to the theory of social capital (Kwok and Gao, 2004), the process of consumers sharing information is a process of actively establishing social relationships and forming social capital. From the perspective of the capitalization process, while the ultimate goal of the sharer is to build their social capital, this must be achieved only if the information they share is perceived as valuable by those they share with. The psychological basis for consumers to share prepaid services at a discount is the recognition that they can avoid loss and bring value to their friends. Scarcity is precious. People generally believe that the scarcer a product or service is, the more valuable it is, and they are more willing to share information about scarcity. Limited-quantity promotions imply both time and quantity restrictions, whereas limited-time promotions imply only time restrictions. So, limited-quantity promotions lead to stronger perceived scarcity (Aggarwal et al., 2011), and consumers’ perceived scarcity will further positively contribute to their willingness to share. Based on this, this study proposes the following hypotheses:

*H1*: When prepaid service is a discount type, limited-quantity promotions can generate a higher willingness to share than limited-time promotions.

*H2*: Perceived scarcity mediates the relationship between limited-quantity promotions (vs. limited time promotions) and willingness to share when prepaid service is a discount type.

### Freebie prepaid service scenarios

The prepaid service mode of the freebie type has a value-added function, and the promotion offers additional benefits to consumers. According to the framing effect theory, in scenarios where individuals are faced with benefits, they usually tend to choose the option with greater certainty to secure the gain (Chatterjee et al., 2014). According to social capital theory (Kwok and Gao, 2004), an individual social capital must be achieved only if he or she believes that the information he or she shares is valuable. In the benefits framework, individuals are more willing to share certainty with others to bring more value to others and enhance their social capital, for both certain and uncertain information. Comparing the characteristics of limited-time and limited-quantity promotions, it is easy to see that limited-time promotions offer greater certainty of promotion opportunities (Wang et al., 2021). Because for consumers, a limited-time promotion means that as long as they participate in the activity within the specified time, they will be able to get the discount. In contrast, limited-quantity promotions are only available for a specific number of products on a first-come, first-served basis, and there is a process of competition with others. The uncertainty of the number of competitors leads to limited-quantity promotions giving consumers a sense of time pressure and uncertainty (Guo et al., 2017). At the same time, it is generally believed that limited-quantity promotions imply a greater crisis of confidence than limited-time promotions because consumers are unaware of the authenticity of changes in limited quantities. Based on this, this study proposes the following hypotheses:

*H3*: When the prepaid service is a freebie, limited-time promotions generate a higher willingness to share than limited-quantity promotions.

*H4*: Perceived opportunity certainty mediates the relationship between limited-time promotions (vs limited-quantity promotions) and willingness to share when prepaid service is a freebie type.

## Materials and methods

### Experimental design

#### Experimental materials

This study selects the typical consumption scenario of tourism and hospitality consumers, that is, checking in a resort hotel, as the research scenario. Resort hotels are generally built near scenic spots and have both accommodation and leisure functions. To attract tourists, resort hotels often launch promotions, especially in the off-season. The general experimental scene we designed is: “Xingxing Hot Spring Hotel” is a hot spring resort hotel located outside a 5A scenic spot in China, about 10 kilometers from the entrance of the scenic spot, which can only be reached by a sightseeing bus at your own expense, the cost of which is CNY50 per person for a round trip. The hot spring hotel has a flat rate of CNY500 per room per visit, which has been maintained since the hotel opened and is widely recognized by tourists. The hotel’s hot spring facilities and overall service are well received by tourists. To promote and attract tourists, the hotel is preparing to launch a “prepaid” activity to attract tourists. There are two prepaid options to choose from.

The first is discounted prepaid service. The specific description is: Consumers can enjoy a 20% discount when they place an order now, that is, they can enjoy the hot spring hotel service with the original price of CNY500 for only CNY400. Consumers can reserve a room in advance at any time within 2 years of placing the order.

The second is a freebie prepaid service. The specific description is: Consumers who place an order and purchase now can enjoy two round-trip free sightseeing bus tickets, that is, for only CNY500, they can enjoy more value-added services than the regular hotel services before. Consumers can reserve a room in advance at any time within 2 years of placing the order.

#### Determination of control variables

Studies have found that a moderate level of promotion is most effective at stimulating consumer behavior (Grewal et al., 1996). Therefore, to avoid the influence of the promotion range on the experiment, this study controlled the promotion range to a medium level, that is, 20% (Alford and Biswas, 2002; Wang et al., 2021). In this study, an online search was conducted to review the pricing of resort-type hotels and calculate the average, which in turn led to the pricing of the resort hotel in the experiment at CNY500 per room per visit. 20% discount equates to a discount of CNY100, which is exactly the cost of two round-trip sightseeing bus tickets. In essence, the effect of the two pre-paid service modes is the same.

The types of promotion purchase restrictions in this study were limited-time promotions and limited-quantity promotions. Longer time horizons can lead to consumer decisions being delayed or even forgotten (Inman and McAlister, 1994). Shorter time limits make consumers too short to think (Sinha, 1994). Similarly, the scope of limited-quantity promotions will also have an influence on consumer behavior. Therefore, taking into account tourism and hospitality practices, we choose a medium level of promotion purchase restriction, that is, 7 days (1 week). Subsequently, we will refer to the research of Aggarwal et al. (2011), and apply the number of limited-quantity promotions equivalent to limited-time promotions for 7 days to this study.

### Experiment 1

#### Experiment design

The purpose of Experiment 1 is to ensure that limited-quantity promotions and limited-time promotions are equally attractive to people. Experiment 1 was divided into two parts, and the data was collected online through the market research company Credamo. Each participant received a reward of CNY0.5. The first time a total of 71 people participated in the experiment, and the second time a total of 63 people participated, both of which are valid data.

#### Procedure and results

In the first experiment, participants were asked to infer the number of limited-quantity promotion, which is equivalent to a limited-time promotion of 7 days (1 week). A total of 71 valid questionnaires were recovered. To ensure the validity of the data, it was necessary to eliminate the influence of extreme numbers on the data, and six outliers were found through SPSS box plots, filling in values greater than or equal to 500. Therefore, this study finally selected the participants whose answers are distributed between 0 and 500, a total of 65 people, of which the average is 124.5, and the mode is 100 (16/65).

The operation of the second experiment was same as the first experiment, but different from the open-ended answer of the first experiment, the answers of the participants needed to choose from six fixed options (20bit/50bit/100 bit/150 bit/200 bit/300 bit). The results of the experiment showed that the majority of participants (17/63) chose 100, with an average value of 117.5. Because in the practice of enterprise promotion activities, the value that is easy to remember is usually taken, the final value chosen for this study is 100, which means that a limited-time promotion of 7 days (1 week) is equivalent to a limited-quantity promotion of 100.

### Experiment 2

#### Experiment design

The purpose of experiment 2 is to examine people’s sharing preferences for promotion purchase restriction types under different prepaid service modes. The experiment used a mixed experimental design of 2 (prepaid mode: discount vs. freebie) × 2 (promotion purchase restrictions: limited-time vs. limited-quantity). Among them, the prepaid mode was a between-group variable, and the promotion purchase restriction was limited to a within-group variable, that was, participants were randomly assigned to two groups (discount vs. freebie), and each participant would face two promotion purchase restrictions and they need to make a selection evaluation. We collected a total of 110 questionnaires through the online market research company Credamo, of which 101 were valid questionnaires (male 44.55%; M age = 30.93, SD = 9.15; *N*_discount_ = 50, *N*_freebie_ = 51). Each participant received a reward of CNY1.

Limited-time promotion was described as: Since the ultimate goal of the hotel is profitability and the hotel’s resources are limited, the hotels will restrict promotions to “prepaid” activities. That is, orders placed within 7 days after this promotion is issued will receive a 20% discount. Limited-quantity promotion was described as: Since the ultimate goal of the hotel is profitability and the hotel’s resources are limited, the hotel will restrict the promotion of “prepaid” activities. That is, the free sightseeing bus tickets for prepaid activities are limited to the first 100 consumers who subscribe after this promotion is issued.

### Procedure

Participants were randomly assigned to one of two groups. The participants were asked to read a piece of material about the prepaid activity firstly, which covered the prepaid modes, the type of promotion purchase restrictions, and some other information about the promotion. After reading the material, participants needed to choose which of the two different types of promotion purchase restriction was more likely to promote their sharing behavior, and then answer open-ended questions to explain the reasons for the choice. In addition, we examined the manipulation of prepaid service modes, types of promotion purchase restrictions involved in the context. And the last were questions on the measurement of demographics, including gender, age, and level of education.

### Results

Manipulation checks. Firstly, the manipulation of the prepaid mode is examined, and the Chi-square test results show that *χ*^2^(1) =5 3.560 (*p* < 0.001), so the control of the prepaid mode in this experiment is successful. Second, the manipulation of the type of promotion purchase restriction needs to be examined. Discount type prepaid group Chi-square test result display that *χ*^2^(1) = 96.078 (*p* < 0.001). Therefore, in this experiment, the prepayment in the discount type is effective to control the promotion purchase limit. Similarly, the Chi-square test results of the prepaid group with the value-added method show that *χ*^2^(1) = 94.302 (*p* < 0.001), so the control of the promotion purchase restriction within the prepaid group in the value-added method is effective in this experiment. In conclusion, the manipulation of the promotion purchase restrictions in this experiment was successful.

Our Chi-square test showed that *χ*^2^(1) = 8.393 (*p* < 0.01), which showed that the interaction of prepaid service mode and promotion purchase restriction had a significant influence on consumers’ willingness to share. Specifically, in the context of discounted prepaid services, 58% of the respondents believed that limited-quantity promotions could promote their sharing willingness and 42% of the respondents held the opposite view. However, the Chi-square test results show that *χ*^2^(1) = 2.560 (*p* = 0.110), which means the difference between the two is not significant, and hypothesis 1 has not been fully verified. In the context of freebie prepaid service, 70.6% of the participants believed that limited-time promotions could promote their sharing behaviors, while only 29.4% of the participants believed that limited-quantity promotion could promote their sharing behaviors, and the Chi-square test result shows *χ*^2^(1) = 17.294 (*p* < 0.001), indicating that the difference between the two is significant, that is, the hypothesis 3 is verified.

### Discussion

Through experiment 2, we found that the interaction of prepaid service mode and promotion purchase restriction will have an influence on consumers’ willingness to share. In a freebie prepaid service context, participants were more likely to share limited-time promotion activities. In the context of discount prepaid services, more participants chose activities with limited-quantity promotion purchase restrictions, but the difference was not significant with limited-time promotion purchase restrictions. Due to the mixed experimental design adopted in this experiment, in which promotion purchase restrictions are within-group factors, the experimental design reduces the influence of the framing effect. Therefore, hypothesis 1 has only been partially verified. In experiment 3, this study will reduce the possible errors through the between-group experimental design and test the hypothesis again.

### Experiment 3

#### Experiment design

The purpose of experiment 3 is to examine the influence of promotion purchase restrictions on consumers’ willingness to share and the psychological mechanism under different prepaid service modes, that is, the mediating path of perceived scarcity and perceived certainty of promotion. This study adopted a between-group experimental design of 2 (prepaid mode: discount type vs. freebie type) × 2 (promotion purchase restriction: limited-time promotion vs. limited-quantity promotion). We randomly assigned all participants to one of four situations. A total of 240 questionnaires were returned for this experiment, with 219 valid questionnaires (male 42.47%; M age = 31.31, SD = 8.50; *N*_discount*limited-time_ = 56, *N*_discount*limited-quantity_ = 56, *N*_freebie*limited-time_ = 55, *N*_freebie*limited-quantity_ = 52). All experimental steps were performed on the online platform Credamo. Each participant received a reward of CNY1.

### Procedure

First, participants were asked to read material about a promotion activity, which covered the way of prepaid service, the type of promotion purchase restriction, and some other information about the promotion. After participants completed reading the material, then we measured their willingness to share, perceived scarcity, and perceived certainty. And a manipulation test was conducted on the prepaid service methods and the types of promotional purchase restrictions faced by the participants in the experimental situation. Finally, participants answered demographic questions including gender, age, education level, occupation, and travel experience. Related variables were measured using the Likert 7-level scale (1 = strongly disagree, 7 = strongly agree). Among them, willingness to share has a total of 3 items, adapted from Zhu et al. (2018), the Cronbach’s *α* was 0.857; perceived scarcity has a total of 3 items, adapted from Lynn and Bogert (1996), Swami and Khairnar (2003), and Wu et al. (2012), the Cronbach’s *α* was 0.863; perceived opportunity certainty has a total of 2 items, adapted from Alavi et al. (2015) and Laran and Tsiros (2013), the Cronbach’s *α* was 0.879. Major measurement items for details in Table 1. As for travel experience, we measured it with a self-assessment item. Namely, please evaluate your travel experience (1 = very little experience, 7 = very rich experience).

**Table 1:** Summary of major measurement items.

Constructs	Items	Adapted from
Willingness to share	I am willing to recommend this prepaid service launched by the hotel to others.	Zhu et al. (2018)
	I am willing to recommend the promotional information of this prepaid service to others.	
	I may talk about this prepaid service with friends around me.	
Perceived scarcity	I think the amount of this prepaid service may be very limited.	Lynn and Bogert (1996), Swami and Khairnar (2003) and Wu et al. (2012)
	I think the prepaid activity will be sold out soon.	
	I think many people will choose to order this prepaid service.	
Perceived opportunity certainty	I’m sure I can finally participate in this prepaid service.	Alavi et al. (2015) and Laran and Tsiros (2013)
	I’m sure I can enjoy the preferential price of this prepaid service.	

### Results

Manipulation checks. Firstly, the manipulation of the prepaid service mode was examined, and the Chi-square test results showed that *χ*^2^(1) = 82.818 (*p* < 0.001), so this experiment was successful in manipulating the prepaid service mode. Secondly, the manipulation of promotion purchase restrictions was examined, the Chi-square test showed *χ*^2^(1) = 188.709 (*p* < 0.001), so this experiment was successful in manipulating the promotion purchase restriction.

**Figure 1:**
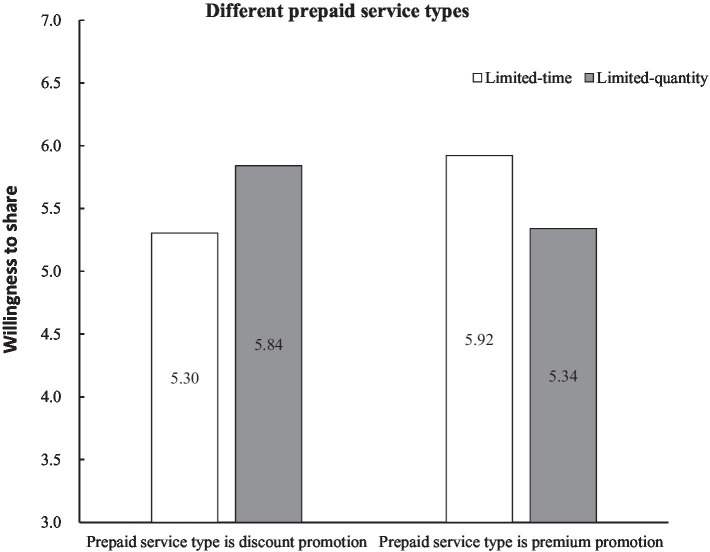
Influence of purchase restrictions on consumers’ willingness to share under different prepaid service types.

Moderating effect test. We first examined the moderating effect of prepaid modes on the relationship between promotion purchase restrictions and consumers’ willingness to share, that was, facing different prepaid service modes, which promotional purchase restrictions could lead to higher sharing willingness. According to the results of multivariate analysis of variance: *F* (1,211) = 24.258, *p* < 0.001, which shows that the interaction of prepaid service mode and promotion purchase restriction types have a significant effect on consumers’ willingness to share. After that, we performed a simple effects analysis. According to Figure 1, under the discounted prepaid service mode, limited-quantity promotion can bring stronger sharing intention than limited-time promotion (*M*_limited-quantity_ = 5.839, *M*_limited-time_ = 5.304, *F* (1,215) = 11.421, *p* = 0.001). Therefore, the hypothesis 1 is verified. Under the freebie prepaid service mode, *M*_limited-time_ = 5.921, *M*_limited-quantity_ = 5.340, *F* (1,215) =12.844, *p* < 0.001. In other words, limited-time promotion can bring higher sharing willingness than limited-quantity promotion, so the hypothesis 3 is verified.

Mediation analysis. In this study, the Bootstrap method (Hayes, 2013) was used for the conditional process model analysis (PROCESS, Model 14, a sample size of 5,000, and the confidence interval was set as 95%) to examine the mediating effects. We also included gender, age, education level, and travel experience as control variables treated in the model.

**Figure 2:**
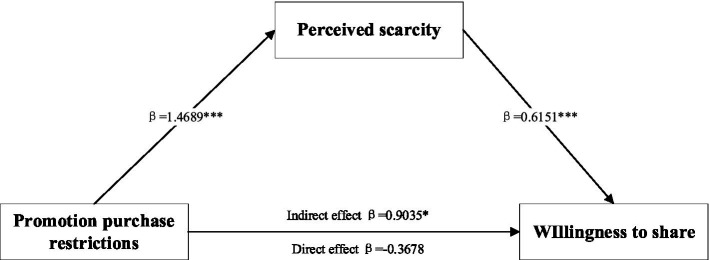
Mediating effect test of perceived scarcity. Note: *p < 0.05, **p < 0.01, and ***p < 0.001.

When the prepaid service mode is a discount type, the results of the independent sample T-test indicate that the perceived scarcity from limited-quantity promotions is greater (*M*_limited-quantity_ = 5.988, *M*_limited-time_ = 4.512, *t* (110) = −11.938, *p* < 0.001). And the results of the test with perceived scarcity as a mediating variable showed that the 95% confidence interval for the Bootstrap test was [0.5349, 1.2405], the interval did not include 0, and the indirect effect *β* = 0.9035. The effect of promotion purchase restrictions on consumers’ willingness to share was no longer significant when mediating variables were controlled (LLCI = −0.8061, ULCI = 0.0686, including 0). It shows that under the prepaid service with discount, the limited-quantity promotion can generate higher willingness to share compared with the limited-time promotion, in which the perceived scarcity plays a mediating role, and the specific mediation path is shown in Figure 2, so the hypothesis 2 is supported.

In addition, we tested whether the certainty of perceived opportunities under discounted prepaid services mediated the relationship between promotion purchase restrictions and willingness to share, and the results showed that the 95% confidence interval of the Bootstrap test was [−0.2879, 0.0738] with an interval including 0, indicating that the certainty of perceived opportunities under discounted prepaid services did not mediate the relationship.

**Figure 3:**
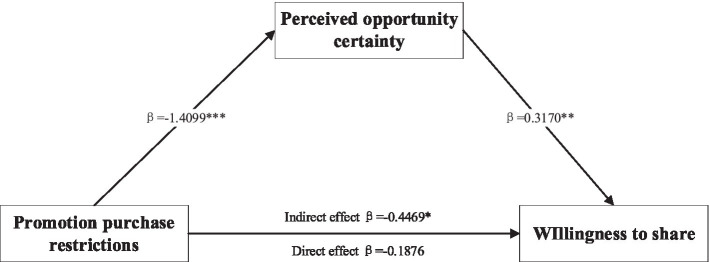
The mediating effect test of perceived opportunity certainty. Note: *p < 0.05, **p < 0.01, and ***p < 0.001.

When the prepaid service mode is a freebie type, according to the independent sample T-test results, it shows that the perceived opportunity brought by the limited-time promotion is more certain (*M*_limited-time_ = 5.200, *M*_limited-quantity_ = 3.799, *t* (105) = 10.390, *p* < 0.001). Second, the results of the test with perceived certainty as the mediating variable showed that the 95% confidence interval of the Bootstrap test was [−0.7914, −0.1696], the interval did not include 0, and the indirect effect *β* = −0.4469, when we controlled the mediating variable, the effect of promotion purchase restriction on consumers’ willingness to share was no longer significant (LLCI = −0.6065, ULCI = 0.2313, including 0). It is suggested that under the prepaid service in the form of a freebie, the limited-time promotion can generate higher willingness to share compared with the limited-quantity promotion, in which the perceived certainty plays a mediating role, and the specific mediation path is shown in Figure 3, so the hypothesis 4 is supported.

In addition, we tested whether perceived scarcity mediates the relationship between promotion purchase restrictions and willingness to share under a freebie prepaid service, and the results showed that the 95% confidence interval for the Bootstrap test was [−0.1840, 0.1988] with an interval including 0, indicating that perceived scarcity does not mediate the relationship under a freebie prepaid service.

### Discussion

Through Experiment 3, we verified that there were differences in consumers’ willingness to share promotion activities when faced with different prepaid service options and promotion purchase restrictions offered by companies. Specifically, when companies offer prepaid services with money-saving features (discounted types), consumers have higher willingness to share in the presence of limited-quantity purchase restrictions (vs. limited-time purchase restrictions), where perceived opportunity certainty plays a mediating role. When companies offer prepaid services with value-added features (freebies types), consumers have a higher willingness to share under limited-time purchase restrictions (vs. limited-quantity purchase restrictions), where perceived scarcity plays a mediating role.

## General discussion and conclusion

### Conclusion

With the increasing popularity of Internet information technology and the influence of COVID-19, the tourism and hospitality are paying more and more attention to the use of prepaid services in promotion activities. From the perspective of framing effect theory, this study focuses on the influence of promotion purchase restrictions on consumers’ willingness to share and its mechanism under different prepaid service modes. During the research process, we proposed and verified the corresponding hypotheses based on the stimulus-organism-response theory. Consumers will form corresponding psychological perceptions (perceived scarcity or perceived certainty) to the stimulation of enterprise promotion activities (combination of prepaid service types and promotion purchase restrictions), and then generate different sharing willingness and behaviors. Through three experiments, we found that in the tourism and hospitality, the prepaid service mode moderates the relationship between promotion purchase restrictions and consumers’ willingness to share. Although the results of Experiment 2 and Experiment 3 are not completely consistent. The difference between the two is mainly reflected in the consumers’ preference for sharing willingness for different promotion purchase restrictions under the discount prepaid service mode. We think this may be related to the experimental design. The two types of promotion purchase restrictions in experiment 2 belong to within group variables, which may lead to a less obvious framing effect for consumers. The design of Experiment 3 has avoided this problem.

In general, when the prepaid service is in the form of a discount, limited-quantity promotions generate higher willingness to share than limited-time promotions, where perceived scarcity plays a mediating role. Because the discounted prepaid service is a loss-reducing promotion, which will reduce the actual amount paid of the buyer. And this prepaid mode is directly linked to the price, it will emphasize how much the consumers spend, when the loss message is highlighted, consumers tend to share valuable information of scarcity to enhance their social capital. The limited-quantity promotion can bring greater perceived scarcity and value compared to the limited-time promotion, so the limited-quantity promotion can better promote the sharing willingness of consumers. When the prepaid service is a freebie, the limited-time promotion can lead to higher sharing willingness than the limited-quantity promotion, where the perceived certainty of the promotion opportunity plays a mediating role. In the prepaid service scenario of the freebie, the benefit information is highlighted, and people tend to choose the option with greater certainty to ensure a higher benefit for the shared information, and limited-time promotions can bring greater certainty than limited-quantity promotions. In this case, limited-time promotions can enhance consumers’ willingness to share.

## Contributions and implications

The theoretical contributions and implications of this study are mainly reflected in the following three aspects. First of all, promotion is one of the most important ways of marketing for companies in the tourism and hospitality. Increasing sales and product promotion are the two most important objectives of promotion. Prepaid service is not only a financial tool, but also a common promotion mode in tourism and hospitality. Previous researches have mainly focused on the influence of promotion modes and related combinations on consumer purchase behavior in the tourism and hospitality (Oruc, 2015). This study mainly focuses on the influence of promotion modes on consumers’ willingness to share, enriches the research literature on promotion, and supplements the researches on consumers’ sharing behavior. Secondly, based on the stimulus-organism-response theory and social capital theory, this study explores the motivation of consumers to share information. While the established literatures have explored consumers’ motivations to provide information as information creators (Berger, 2014), few literatures have explored consumers’ motivations to disseminate information as information distributors. And this study is an exploration of the psychological mechanisms of consumer sharing behavior. Finally, this study verifies the mediating role of perceived scarcity and perceived opportunity certainty on the relationship between promotion purchase restrictions and sharing willingness under different prepaid service modes. Our study provides a new research perspective on the interaction and application of promotion modes and promotion purchase restrictions.

Our findings also provide important marketing implications for the tourism and hospitality. First of all, enterprises should pay attention to the refined management of promotions, not blindly carry out promotion activities, and should formulate their promotion combination modes based on the industry characteristics and company’s resources. Second, enterprises should provide differentiated prepaid service modes and promotion purchase restrictions at different times and environments to improve promotion efficiency. Prepaid services such as discounts are paired with limited-time promotions rather than limited-quantity promotions. Finally, consumers’ willingness to share promotions is mainly based on perceived scarcity or perceived opportunity certainty. Therefore, the marketers of enterprises should pay attention to consumers’ emotion and psychological state, and manipulate consumers’ perceived scarcity and perceived opportunity certainty through appropriate communication, to obtain better promotion effects. For the government, it is recommended to strengthen the management of enterprise’s promotion activities, especially the authenticity and transparency of information, so that consumers can truly enjoy the benefits of prepaid services.

## Limitations and future research directions

This study has certain limitations, and follow-up researches can be further explored and improved. First, the experiment data in this study was mainly derived from online questionnaires and not from real consumer behaviors in real scenarios in the tourism and hospitality. Future studies may consider using more realistic experiment methods or seek to collaborate with companies offering promotions to obtain real consumer behavior data for further researches. Second, this study only discusses the effects of limited-time promotion purchase restrictions and limited-quantity promotion purchase restrictions on consumers’ willingness to share. The influential role of other types of promotion purchase restrictions, such as membership restrictions, monetary restrictions, and use period restrictions, may be considered in the future. At the same time, this study only considers the effects of limited-time promotion and limited-quantity promotion on consumers’ willingness to share separately. Future researches can also explore the influence of limited-time and limited-quantity use simultaneously on sharing willingness. Third, this study only focuses on the influence of prepaid services on consumer behavior. In the future, other scholars can also study the influence and mechanism of other financial tools on consumer behavior. Finally, consumer responses to scarcity promotions and prepaid service modes may also be influenced by other factors, such as consumer personality, product types, discount strength, gift types, etc. The effects of other boundary conditions may be considered in the future, and other psychological mechanisms of action may be further explored.

## Data availability statement

The raw data supporting the conclusion of this article will be made available by the authors, without undue reservation.

## Ethics statement

Ethical review and approval were not required for the study on human participants in accordance with the local legislation and institutional requirements. Written informed consent from the patients/participants or patients/participants legal guardian/next of kin was not required to participate in this study in accordance with the national legislation and the institutional requirements.

## Author contributions

All authors listed have made a substantial, direct, and intellectual contribution to the work, and approved it for publication.

## Conflict of interest

The authors declare that the research was conducted in the absence of any commercial or financial relationships that could be construed as a potential conflict of interest.

## Publisher’s note

All claims expressed in this article are solely those of the authors and do not necessarily represent those of their affiliated organizations, or those of the publisher, the editors and the reviewers. Any product that may be evaluated in this article, or claim that may be made by its manufacturer, is not guaranteed or endorsed by the publisher.
